# Scheelite-type NaDy(WO_4_)_2_

**DOI:** 10.1107/S1600536809052271

**Published:** 2009-12-12

**Authors:** Dan Zhao, Feifei Li, Wendan Cheng, Hao Zhang

**Affiliations:** a Department of Physics and Chemistry, Henan Polytechnic University, Jiaozuo, Henan 454000, People’s Republic of China; b State Key Laboratory of Structural Chemistry, Fujian Institute of Research on the Structure of Matter, Chinese Academy of Sciences, Fuzhou, Fujian 350002, People’s Republic of China

## Abstract

The title compound sodium dysprosium(III) bis­[tungs­tate(VI)], NaDy(WO_4_)_2_, has been synthesized under high temperature solution growth (HTSG) conditions in air. The compound crystallizes with the scheelite structure and is composed of isolated WO_4_ tetra­hedra (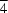 symmetry) with one set of bond lengths and distorted [(Na/Dy)O_8_] dodeca­hedra (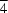 symmetry; occupancy ratio Na:Dy = 1:1) with two sets of bond lengths.

## Related literature

For the structures, properties and applications of alkali rare-earth bis-tungstates with general formula *ARE*(WO_4_)_2_ (*A* = alkali metal, *RE* = rare-earth metal), see: Perets *et al.* (2007 ▶); Han *et al.* (2002 ▶); Huang *et al.* (2006 ▶); Li *et al.* (1990 ▶). For the scheelite (CaWO_4_) structure, see: Sillen & Nylander (1943 ▶).

## Experimental

### 

#### Crystal data

NaDy(WO_4_)_2_
*M* *_r_* = 681.19
Tetragonal, 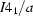

*a* = 5.2545 (5) Å
*c* = 11.4029 (15) Å
*V* = 314.83 (6) Å^3^
*Z* = 2
Mo *K*α radiation
μ = 48.27 mm^−1^
*T* = 298 K
0.10 × 0.10 × 0.08 mm

#### Data collection

Rigaku Mercury70 diffractometer
Absorption correction: multi-scan (*SADABS*; Sheldrick, 1997 ▶) *T*_min_ = 0.263, *T*_max_ = 1.000
1128 measured reflections
181 independent reflections
143 reflections with *I* > 2σ(*I*)
*R*_int_ = 0.053

#### Refinement

*R*[*F* ^2^ > 2σ(*F* ^2^)] = 0.024
*wR*(*F* ^2^) = 0.056
*S* = 0.87
181 reflections
15 parameters
Δρ_max_ = 1.55 e Å^−3^
Δρ_min_ = −1.40 e Å^−3^

### 

Data collection: *CrystalClear* (Rigaku, 2000 ▶); cell refinement: *CrystalClear*; data reduction: *CrystalClear*; program(s) used to solve structure: *SHELXS97* (Sheldrick, 2008 ▶); program(s) used to refine structure: *SHELXL97* (Sheldrick, 2008 ▶); molecular graphics: *DIAMOND* (Brandenburg, 2004 ▶); software used to prepare material for publication: *SHELXTL* (Sheldrick, 2008 ▶).

## Supplementary Material

Crystal structure: contains datablocks I, global. DOI: 10.1107/S1600536809052271/wm2287sup1.cif

Structure factors: contains datablocks I. DOI: 10.1107/S1600536809052271/wm2287Isup2.hkl

Additional supplementary materials:  crystallographic information; 3D view; checkCIF report

## Figures and Tables

**Table 1:** Selected bond lengths (Å)

(Na/Dy)1—O1^i^	2.457 (7)
(Na/Dy)1—O1	2.471 (7)
W1—O1	1.785 (7)

Symmetry code: (i) 
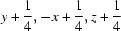
.

**Fig. 1.**
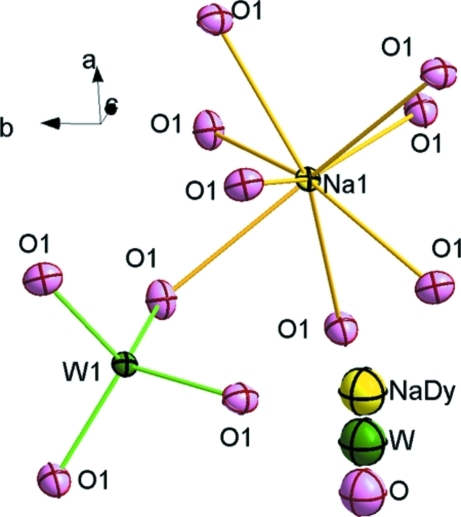
Part of the structure of NaDy(WO4)2 showing the labelling of the atoms (displacement ellipsoids are drawn at the 50% probability level).

**Fig. 2.**
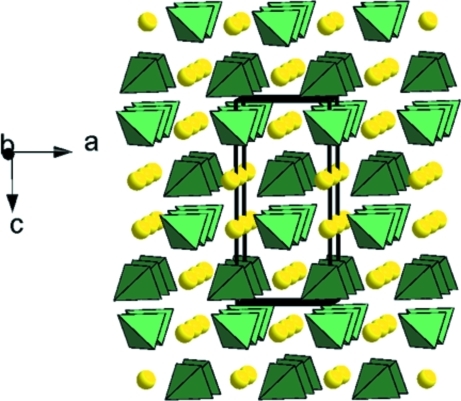
View of the crystal structure of NaDy(WO4)2 (WO4 tetrahedra are shaded in sea-green; Na/Dy atoms are drawn as yellow balls).

## supplementary crystallographic information

### Comment

In the past years an increasing interest in the synthesis and characterization of rare-earth double tungstate(VI) crystals with general formula *ARE*(WO_4_)_2_ (*A* = alkali metal, *RE* = rare-earth metal) has been observed due to their interesting magnetic, electric and optical properties (Perets *et al.*, 2007; Huang *et al.*, 2006; Li *et al.*, 1990; Han *et al.*, 2002). These compounds are attractive solid-state laser host materials because of their large rare-earth ion admittance. Most of these crystals have tetragonal symmetry and crystallize with the scheelite structure (CaWO_4_) in space group *I*4_1_/*a* (Sillen & Nylander, 1943). In the title structure, the Ca^2+^ position of the original CaWO_4_ structure is statistically occupied by Na^+^ and Dy^3+^ ions in an 1:1 ratio. The crystal structure of NaDy(WO_4_)_2_ is composed of a two-direction packing of isolated WO_4_ tetrahedra interconnected by distorted [(Na/Dy)O_8_] dodecahedra, as shown in Fig. 2.

### Experimental

Single crystal of NaDy(WO_4_)_2_ were prepared by a high temperature solution reaction, using analytical reagents of Dy_2_O_3_, Na_2_CO_3_ and WO_3_ in the molar ratio of Na: Dy: W = 8:1:10. The starting mixture was finely ground in an agate mortar to ensure the best homogeneity and reactivity, and then transferred to a platinum crucible to be heated at a temperature of 773 K for 8 h. The sintered product was reground and continuously heated at 1273 K for 20 h, cooled to 673 K at a rate of 4 K/h, and then quenched to room temperature. A few light yellow and prismatically shaped crystals of the title compound were obtained.

### Refinement

The Na1 and Dy1 atoms are in a substitutional-type disorder in the structure. Therefore the atomic position and anisotropic displacement parameters of Na1 and Dy1 atoms were constrained to be identical. In the initial least-squares refinement, the occupancy factors of Na1 and Dy1 atoms were set to be free. The results show that the occupancy factors were close to 1:1, *viz* Na1: Dy1 = 0.50273: 0.49727, and were eventually fixed in a 1:1 ratio. The highest peak in the final difference electron density map is 1.55 e/Å^3^ from the W1 site, and the deepest hole is -1.40 e/Å^3^ from the Na1/Dy1 site.

### Crystal data

**Table:**

NaDy(WO_4_)_2_	*D*_x_ = 7.186 Mg m^−^^3^
*M**_r_* = 681.19	Mo *K*α radiation, λ = 0.71073 Å
Tetragonal, *I*4_1_/*a*	Cell parameters from 405 reflections
Hall symbol: -I 4ad	θ = 3.6–27.5°
*a* = 5.2545 (5) Å	µ = 48.27 mm^−^^1^
*c* = 11.4029 (15) Å	*T* = 298 K
*V* = 314.83 (6) Å^3^	Prism, light yellow
*Z* = 2	0.10 × 0.10 × 0.08 mm
*F*(000) = 578	

### Data collection

**Table:**

Rigaku Mercury70 diffractometer	181 independent reflections
Radiation source: fine-focus sealed tube	143 reflections with *I* > 2σ(*I*)
graphite	*R*_int_ = 0.053
Detector resolution: 14.6306 pixels mm^-1^	θ_max_ = 27.5°, θ_min_ = 4.3°
ω scans	*h* = −6→6
Absorption correction: multi-scan (*SADABS*; Bruker, 1997)	*k* = −6→6
*T*_min_ = 0.263, *T*_max_ = 1.000	*l* = −14→13
1128 measured reflections	

### Refinement

**Table:**

Refinement on *F*^2^	Primary atom site location: structure-invariant direct methods
Least-squares matrix: full	Secondary atom site location: difference Fourier map
*R*[*F*^2^ > 2σ(*F*^2^)] = 0.024	*w* = 1/[σ^2^(*F*_o_^2^) + (0.0045*P*)^2^] where *P* = (*F*_o_^2^ + 2*F*_c_^2^)/3
*wR*(*F*^2^) = 0.056	(Δ/σ)_max_ < 0.001
*S* = 0.87	Δρ_max_ = 1.55 e Å^−^^3^
181 reflections	Δρ_min_ = −1.40 e Å^−^^3^
15 parameters	Extinction correction: *SHELXL97* (Sheldrick, 2008), Fc^*^=kFc[1+0.001xFc^2^λ^3^/sin(2θ)]^-1/4^
0 restraints	Extinction coefficient: 0.0295 (17)

### Special details

**Table:**

Geometry. All e.s.d.'s (except the e.s.d. in the dihedral angle between two l.s. planes) are estimated using the full covariance matrix. The cell e.s.d.'s are taken into account individually in the estimation of e.s.d.'s in distances, angles and torsion angles; correlations between e.s.d.'s in cell parameters are only used when they are defined by crystal symmetry. An approximate (isotropic) treatment of cell e.s.d.'s is used for estimating e.s.d.'s involving l.s. planes.
Refinement. Refinement of *F*^2^ against ALL reflections. The weighted *R*-factor *wR* and goodness of fit *S* are based on *F*^2^, conventional *R*-factors *R* are based on *F*, with *F* set to zero for negative *F*^2^. The threshold expression of *F*^2^ > σ(*F*^2^) is used only for calculating *R*-factors(gt) *etc*. and is not relevant to the choice of reflections for refinement. *R*-factors based on *F*^2^ are statistically about twice as large as those based on *F*, and *R*- factors based on ALL data will be even larger.

### Fractional atomic coordinates and isotropic or equivalent isotropic displacement parameters (Å^2^)

**Table:**

	*x*	*y*	*z*	*U*_iso_*/*U*_eq_	Occ. (<1)
Na1	0.5000	−0.2500	0.1250	0.0068 (4)	0.50
Dy1	0.5000	−0.2500	0.1250	0.0068 (4)	0.50
W1	0.0000	0.2500	0.1250	0.0091 (4)	
O1	0.2419 (14)	0.0977 (13)	0.0404 (6)	0.0187 (16)	

### Atomic displacement parameters (Å^2^)

**Table:**

	*U*^11^	*U*^22^	*U*^33^	*U*^12^	*U*^13^	*U*^23^
Na1	0.0090 (6)	0.0090 (6)	0.0024 (8)	0.000	0.000	0.000
Dy1	0.0090 (6)	0.0090 (6)	0.0024 (8)	0.000	0.000	0.000
W1	0.0098 (4)	0.0098 (4)	0.0076 (6)	0.000	0.000	0.000
O1	0.024 (5)	0.017 (4)	0.015 (3)	0.001 (3)	0.003 (3)	0.001 (3)

### Geometric parameters (Å, °)

**Table:**

(Na/Dy)1—O1^i^	2.457 (7)	(Na/Dy)1—O1^vii^	2.471 (7)
(Na/Dy)1—O1^ii^	2.457 (7)	(Na/Dy)1—O1	2.471 (7)
(Na/Dy)1—O1^iii^	2.457 (7)	W1—O1^viii^	1.785 (7)
(Na/Dy)1—O1^iv^	2.457 (7)	W1—O1^ix^	1.785 (7)
(Na/Dy)1—O1^v^	2.471 (7)	W1—O1	1.785 (7)
(Na/Dy)1—O1^vi^	2.471 (7)	W1—O1^x^	1.785 (7)
			
O1^i^—(Na/Dy)1—O1^ii^	79.7 (3)	O1^vi^—(Na/Dy)1—O1^vii^	98.76 (12)
O1^i^—(Na/Dy)1—O1^iii^	126.1 (2)	O1^i^—(Na/Dy)1—O1	68.80 (16)
O1^ii^—(Na/Dy)1—O1^iii^	126.1 (2)	O1^ii^—(Na/Dy)1—O1	76.3 (3)
O1^i^—(Na/Dy)1—O1^iv^	126.1 (2)	O1^iii^—(Na/Dy)1—O1	73.31 (14)
O1^ii^—(Na/Dy)1—O1^iv^	126.1 (2)	O1^iv^—(Na/Dy)1—O1	152.4 (3)
O1^iii^—(Na/Dy)1—O1^iv^	79.7 (3)	O1^v^—(Na/Dy)1—O1	98.76 (12)
O1^i^—(Na/Dy)1—O1^v^	152.4 (3)	O1^vi^—(Na/Dy)1—O1	98.76 (12)
O1^ii^—(Na/Dy)1—O1^v^	73.31 (14)	O1^vii^—(Na/Dy)1—O1	134.1 (3)
O1^iii^—(Na/Dy)1—O1^v^	68.80 (16)	O1^viii^—W1—O1^ix^	107.0 (2)
O1^iv^—(Na/Dy)1—O1^v^	76.3 (3)	O1^viii^—W1—O1	114.6 (4)
O1^i^—(Na/Dy)1—O1^vi^	73.31 (14)	O1^ix^—W1—O1	107.0 (2)
O1^ii^—(Na/Dy)1—O1^vi^	152.4 (3)	O1^viii^—W1—O1^x^	107.0 (2)
O1^iii^—(Na/Dy)1—O1^vi^	76.3 (3)	O1^ix^—W1—O1^x^	114.6 (4)
O1^iv^—(Na/Dy)1—O1^vi^	68.80 (16)	O1—W1—O1^x^	107.0 (2)
O1^v^—(Na/Dy)1—O1^vi^	134.1 (3)	W1—O1—Dy1^ii^	131.4 (3)
O1^i^—(Na/Dy)1—O1^vii^	76.3 (3)	W1—O1—(Na/Dy)1^ii^	131.4 (3)
O1^ii^—(Na/Dy)1—O1^vii^	68.80 (16)	W1—O1—(Na/Dy)1	120.8 (3)
O1^iii^—(Na/Dy)1—O1^vii^	152.4 (3)	Dy1^ii^—O1—(Na/Dy)1	103.7 (3)
O1^iv^—(Na/Dy)1—O1^vii^	73.31 (14)	(Na/Dy)1^ii^—O1—(Na/Dy)1	103.7 (3)
O1^v^—(Na/Dy)1—O1^vii^	98.76 (12)		

Symmetry codes: (i) *x*, *y*−1/2, −*z*; (ii) −*x*+1, −*y*, −*z*; (iii) *y*+1/4, −*x*+1/4, *z*+1/4; (iv) −*y*+3/4, *x*−3/4, *z*+1/4; (v) *y*+3/4, −*x*+1/4, −*z*+1/4; (vi) −*y*+1/4, *x*−3/4, −*z*+1/4; (vii) −*x*+1, −*y*−1/2, *z*; (viii) −*x*, −*y*+1/2, *z*; (ix) −*y*+1/4, *x*+1/4, −*z*+1/4; (x) *y*−1/4, −*x*+1/4, −*z*+1/4.
